# Cardiomyopathy and Sudden Cardiac Death Among the Athletes in Developing Countries: Incidence and Their Prevention Strategies

**DOI:** 10.7759/cureus.35612

**Published:** 2023-02-28

**Authors:** Mohamed Rage, Mohamed Mohamed, Mohammed A Nor, Nawal Abdi, Jerry J Akplor, Siva Naga S Yarrarapu, Parth Shah, Beshoy Iskander

**Affiliations:** 1 Internal Medicine, Wuhan University, Wuhan, CHN; 2 Internal Medicine, Norman Bethune Health Science Center of Jilin University, Jilin, CHN; 3 Internal Medicine, Capital Medical University, Beijing, CHN; 4 Faculty of Medicine, Hebei North University, Zhangjiakou, CHN; 5 Internal Medicine, Monmouth Medical Center/Rutgers University, Long Branch, USA; 6 Hospital Medicine, Tower Health Medical Group, West Reading, USA; 7 Internal Medicine, Bon Secours Mercy Health, St. Elizabeth Youngstown Hospital (NEOMED), Ohio, USA

**Keywords:** cardiac arrest, sudden cardiac death, developing countries, athletes, hypertrophic cardiomyopathy, cardiomyopathies

## Abstract

The incidence of cardiomyopathy in athletes contributes significantly to the public health burden in developing countries. Most effective management strategies primarily rely on the modification of risk factors, and it is less expensive compared to other advanced investigations. Moreover, limited data is available concerning the incidence of adverse events including cardiac arrest and the strategies to prevent them, especially in this population subset. Therefore, devising preventative strategies that can easily be implemented in athletes and provide a cost-effective approach is warranted.

We aim to discuss the incidence of major adverse cardiac events in athletes with cardiomyopathies and their associated risk factors and to evaluate the various strategies proposed to prevent the progression of cardiomyopathy in this population, with the initial hypothesis that the treatment of these pathologies poses a substantial challenge in this population. With regard to methodology, this is a narrative review. Search terms were described using the Population, Exposure, and Outcome (PEO) framework. A comprehensive search strategy was used to screen and identify any relevant literature in the PubMed and Google Scholar databases. This was done in accordance with the Preferred Reporting Items for Systematic Review and Meta-Analysis (PRISMA) protocol. Four studies were identified in the final analysis. The incidence of sudden cardiac arrest varied between 0.3% and 0.33% among the athletes affected with cardiomyopathies.

Routine and pre-participation screening has shown success in reducing the incidence of sudden cardiac death in athletes as a result of undiagnosed cardiomyopathies. Supervised exercise regimes have been proposed to reduce the incidence of cardiomyopathy in athletes. Beyond identification strategies, the prevention of cardiomyopathies revolves around the modification of risk factors. To conclude, the challenges athletes face, suffering from cardiomyopathy, have been an ongoing issue with unexpected cardiac arrest as the end result. Despite the decreased incidence of cardiomyopathies observed in athletes, the challenge in diagnosis can result in catastrophic outcomes, especially in developing countries. Therefore, adopting prevention strategies can have a profound impact on the identification and management of these pathologies.

## Introduction and background

Cardiomyopathies represent a heterogeneous group of pathologies that are characterized by a vast number of structural and functional alterations of a patient’s myocardium [1]. Cardiomyopathy, therefore, has several classifications and can be separated into three primary categories: genetic, mixed, or acquired, and phenotypically into hypertrophic, dilated, and restrictive patterns [2]. The most common primary cardiomyopathy is hypertrophic cardiomyopathy, with studies estimating a prevalence between 0.16% and 0.29% in the general adult population [3-5]. The most effective strategy to prevent cardiomyopathy lies in the modification of risk factors. Although the literature describes several potential risk factors for the development of cardiomyopathy, the most widely accepted predispositions are coronary artery disease and other atherosclerotic vascular diseases, hypertension, diabetes, renal insufficiency, obesity, and a family history of cardiomyopathy [6]. Risk can be modified via lifestyle changes and/or therapeutic interventions, including angiotensin-converting enzyme inhibitors, beta-blockers, aspirin, and statin therapy [7].

However, despite the low prevalence of these pathologies, the burden of cardiomyopathy is significant. This public health burden lies in the poor prognosis, with studies suggesting that cardiomyopathies account for 51% of all heart transplants, 33% of defibrillator implantations, 38% of mechanical circulatory supports, and 11.3% of hospitalizations for heart failure [8].

Hypertrophic cardiomyopathy is of important public health concern among athletes in recent years. Although cardiomyopathies are widely discussed in the current literature, the research fails to specifically discuss the incidence of these pathologies and strategies to prevent them in developing countries, particularly among athletes. Therefore, this literature review aims to review and discuss the available literature that focuses on cardiomyopathies in this population.

## Review

Methods

Narrative Review

This narrative review and its objectives were focused on the incidence of major adverse cardiac events in athletes with cardiomyopathy and their associated risk factors in developing countries. This article aims to evaluate the various strategies proposed to prevent the progression of cardiomyopathy in this population, with the initial hypothesis that the treatment of these pathologies poses a substantial challenge in this population.

Search Strategy

A Population, Exposure, and Outcome (PEO) framework was, therefore, devised to identify the relevant search terms that were adopted in the search strategy, with PubMed and Google Scholar databases being screened for relevant literature. Table 1 provides an overview of this framework and the keywords identified.

**Table 1:** PEO framework for use in the search strategy PEO: Population, Exposure, and Outcome.

PEO framework	
Population	Athletes, professional athletes, sporting, sports, and developing countries
Exposure	Cardiomyopathies, cardiomyopathy, and hypertrophic cardiomyopathy
Outcome	Treatment, patient outcome, symptom reduction, risk factor modification, sudden cardiac death, and cardiac arrest

Inclusion Criteria

Studies performed on athletes, studies completed in developing countries, full-text articles, observational studies, and randomized controlled trials were included in this study.

Exclusion Criteria

Non-English studies and in-vivo studies were excluded from the review.

Data Extraction

In accordance with the PRISMA (Preferred Reporting Items for Systematic Review and Meta-Analysis) protocol, two independent reviewers identified relevant abstracts from the literature based on the search strategy in separate databases. Duplicates were then removed, and the full text was assessed for eligibility based on the inclusion and exclusion criteria. A third reviewer verified the shortlisted papers and resolved any conflicts. The entire process was completed manually, and no software was utilized for the data extraction.

Results

In this review, we have searched through PubMed and Google Scholar databases and identified four studies.(Table 2). These studies were from diverse regions and provided significant results on the association of cardiomyopathy in athletes.

**Table 2:** A review of studies demonstrating the incidence of cardiomyopathies of athletes in developing countries and their prevention strategies DCM: Dilated cardiomyopathy; HCM: Hypertrophic cardiomyopathy; IDCM: Idiopathic dilated cardiomyopathy; SCA: Sudden cardiac arrest; SCD: Sudden cardiac death.

Study name, year	Study design	Age (years); Gender (number included in the study/total sample)	Study sample	Initial presentation and/or study objectives	Main findings	Conclusion
Pelliccia et al., 2020 [17]	Prospective cohort	31 (median); Male (81/88)	88 athletes diagnosed with HCM	Most athletes were asymptomatic (n = 67). Physical examination was unremarkable in most participants (n = 80); eight participants had systolic ejection murmur.	During the follow-up period of 7 ± 5 years, two participants suffered sudden cardiac arrest or death (0.3% per year), and 19 reported symptoms such as syncope, palpitation, chest pain, and dyspnea.	In low-risk HCM athletes, participation in competitive sports events was not associated with increased risk for major cardiac events or clinical worsening compared with reduced or withdrawal exercise programs and sports (p = 0.264). This may not be true in patients with more severe HCM phenotypes.
Tchanana et al., 2020 [18]	Study 1: Prospective cohort; Study 2: Cross-sectional	56 (median); Male (49.5%)	86,189 inhabitants of sub-Saharan Africa	Determining the incidence of sports-related sudden cardiac death	The age-adjusted incidence of SCA in the general population was 33.6 (95% CI: 22.2-44.9) cases per 100,000 persons per year. Given the prevalence of recreational sport athletes of 69.0%, there was one sports-related cardiac arrest in this population out of 59,452 athletes per year, representing 1.7 (95% CI: 0.2-12.0) cases per 100,000 athletes per year.	Limitations to the effective pre-participation screening of athletes: limited availability of ECG machines and the cost of examination; insufficient number of physicians qualified in sports; and limited access to medical care.
Schmied et al., 2013 [12]	Cross-sectional	18.6 (mean); Male (100%)	210 Gabonian football players	Pre-participation cardiovascular screening using history, physical exam, ECG, and echo in an African setting to reduce SCD	9% reported atypical chest discomfort not related to exercise. The family history of SCD is present in 17%. ECG showed large proportions of “training-related” abnormalities including ST-segment elevation (early repolarization) in precordial leads in 71.4% and isolated increase in R/S-wave voltage in 55.2%. 12.4% showed “training-unrelated” abnormalities (indicating potential expression of cardiac disease) including inverted T-waves, left atrial enlargement, and deep Q-waves.	12.4% showed “training-unrelated” ECG abnormalities and underwent additional testing and periodical follow-up. Structural abnormalities were found in the minority (5%), including HCM. No ARVC or DCM was found. Pre-participation CV screening is efficient to identify (or raise suspicion) CV abnormalities in native African athletes.
Bailly et al., 2019 [16]	Cross-sectional	33 (median)	82 asymptomatic family members (55 first-degree and 27 second-degree relatives) of probands with IDCM	Asymptomatic	No asymptomatic family members were identified with features of DCM or presymptomatic DCM. Possible presymptomatic DCM was identified by abnormalities on the echocardiogram in three families (four first-degree relatives). Possible presymptomatic DCM was identified on the basis of cardiac conduction abnormality in eight families (nine first-degree and two second-degree relatives).	Screening for IDCM should include a three-generation family history and clinical screening of all first-degree relatives. As IDCM has an age-related penetrance, at-risk family members should receive follow-up for screening to assess symptoms and signs of IDCM. Genetic testing would potentially identify family members at high risk, who would benefit from screening.

Qualitative analysis of the included studies suggests that athletes are less likely to develop cardiomyopathies as they do not often present with the risk factors for these pathologies. However, in athletes when cardiomyopathy develops, diagnosis presents a significant challenge, and this often results in catastrophic outcomes, including sudden cardiac arrest [9].

Hypertrophic Cardiomyopathy and Sudden Cardiac Death Among Athletes in Developing Countries

Hypertrophic cardiomyopathy represents the primary cause of sudden cardiac death among athletes under the age of 35 years. The incidence of sudden cardiac arrest varied between 0.3% and 0.33% among this population subset. However, diagnosis of this condition and subsequent management remain a significant challenge plaguing healthcare providers as the characteristic hypertrophy of the left ventricle observed in cardiomyopathies mimics the physiological left ventricular hypertrophy in response to exercise [10]. In athletes where hypertrophic cardiomyopathy has been diagnosed, current guidelines recommend a precautionary disqualification from all competitive sports [11].

Prevention Strategies

The main prevention strategies identified in the current literature revolve around cardiac screening, whether that be regular screening or pre-participation screening. There is substantial evidence that implementing this strategy in developing countries reduces the incidence of sudden cardiac death in athletes as a result of undiagnosed cardiac disease. Supervised exercise regimes have also been proposed.

Discussion

Cardiac screening represents one of the most prevalently adopted strategies to reduce the incidence of debilitating cardiomyopathy and sudden cardiac death across the globe, even in developing countries. A study by Schmied et al. performed a pre-participation cardiovascular screening in an African setting to reduce sudden cardiac death in 210 male Gabonian football players. Atypical chest discomfort and oppression were observed in 19 players (9%). Moreover, several different abnormalities were observed following ECG, including ST-segment elevation in precordial leads (150, 71.4%), isolated increase in R/S-wave voltage (116, 55.6%), inverted T-waves (10, 4.8%), left atrial enlargement (8.4%), and deep Q-waves (3, 1.4%). Collectively, 12% of the native African athletes screened in this study showed ECG abnormalities that were unrelated to training and warranted further testing and periodical follow-up. Hence, utilizing EKG in cardiac screening demonstrates its efficiency in identifying and raising suspicion about cardiovascular abnormalities in this population, potentially reducing the incidence of debilitating cardiomyopathy and sudden cardiac death [12].

Sudden cardiac death is a potential outcome in all patients with cardiac disease, and those with structural heart disease and worsened ventricular dysfunction are at an increased risk of this outcome [13]. A review by Vora et al. explored the strategies devised for the prevention of sudden cardiac death in athletes, sportspersons, and marathoners in India. Two primary strategies were discussed: pre-participation screening and a supervised, graded exercise regime. The pre-participation screening revolves around identifying any at-risk athletes through a comprehensive assessment of their history and a physical examination. In athletes of concern, an ECG and an echocardiogram may be recommended. The supervised, graded exercise regimen, on the other hand, is targeted toward older uninitiated individuals that are participating in enduring sporting activities, such as marathons. Under this strategy, high-risk individuals are identified in a similar manner to the pre-participation screening and undergo exercise testing alongside a supervised exercise program [14].

The hypothesis that cardiac screening mitigates the risk of sudden cardiac death in athletes was comprehensively investigated in a systematic review of Middle Eastern and African studies. Here, Hallak et al. investigated cardiac pre-participation screening methods in this population. The findings demonstrate that the prevalence of sudden cardiac death-related abnormalities, including hypertrophic cardiomyopathy, ranged from 0.47% to 4.29% and that pre-participation screening is effective in identifying these abnormalities. It was also observed that African athletes have a high false-positive rate when compared to Arab and Caucasian athletes with respect to interpreting the EKGs [15].

Bailly et al. also proposed that family history assessments alongside clinical screening may be beneficial in identifying athletes with idiopathic dilated cardiomyopathy in Johannesburg, South Africa [16]. Pelliccia et al. assessed the incidence of cardiovascular events in 88 adult athletes with hypertrophic cardiomyopathy who engaged in long-term exercise programs and competitive sports, finding sudden cardiac arrest in two athletes (0.3% per year) and symptomatic disease in 19 athletes (22%), including syncope, palpitations, chest pain, and dyspnea [17]. A further study conducted by Tchanana et al. investigated the incidence of recreational sports-related sudden cardiac arrest in 86,189 participants over the age of 12 years in a general African population. Sudden cardiac arrest occurred in 0.33% (288 persons). The associated cardiovascular diseases among these persons were hypertension (22.2%), diabetes (11.1%), heart failure (14.8%), dilated cardiomyopathy (7.4%), and myocardial infarction (7.4%) [18]. Hypertension, diabetes, and heart failure are all risk factors for the progression of cardiac disease; therefore, modifying these risk factors is crucial for athletes and sporting participants to prevent the occurrence of catastrophic outcomes, including sudden cardiac arrest.

As reported in the study by Tchanana et al., the biggest limitations to effective pre-participation screening in many African countries include limited availability of ECG machines, the cost of examination, inadequate number of physicians certified in sports medicine, and limited access to health care with few specialized sports medicine clinics, all of which might hinder the disqualification of athletes with hypertrophic cardiomyopathy from the sports competition [18].

## Conclusions

Despite the decreased incidence of cardiomyopathy among athletes in developing countries, the challenge in diagnosis can result in catastrophic outcomes. Therefore, adopting prevention strategies, including pre-participation screening and regular cardiac screening utilizing cost-effective measures like EKG, can have a profound impact on the identification and management of these pathologies. Family history assessments alongside clinical screening may be beneficial in identifying athletes with idiopathic dilated cardiomyopathy. Beyond identification strategies, preventing the progression of cardiac disease revolves around the modification of risk factors.
